# Kinetic Parameters of Phosphorus Uptake as a Function of Cationic Metal Supply in Cotton

**DOI:** 10.3390/plants15081215

**Published:** 2026-04-15

**Authors:** Elcio Ferreira Santos, Ana Beatriz Pires Silva, Moacir de Souza Silva, Silvana de Paula Quintão Scalon, José Lavres

**Affiliations:** 1 Instituto Federal de Mato Grosso do Sul, Nova Andradina 79750-000, MS, Brazil; 2 Faculdade de Ciências Agrárias, Universidade Federal da Grande Dourados, Dourados 79825-070, MS, Brazil; agro.anabeatriz@gmail.com (A.B.P.S.); moacir.silva@novaandradina.org (M.d.S.S.); silvanascalon@ufgd.edu.br (S.d.P.Q.S.); 3 Centro de Energia Nuclear na Agricultura, Piracicaba 13416-000, SP, Brazil; jlavres@usp.br

**Keywords:** *Gossypium hirsutum* L., Vmax, Km, Cmin, uptake dynamics

## Abstract

Phosphorus (P) availability is currently a limiting factor for agricultural production, especially in tropical soils, and its interaction with cationic micronutrients can significantly affect physiological efficiency and nutrient uptake by plants. Therefore, this study aimed to evaluate the uptake kinetic parameters described by the Michaelis–Menten model (Vmax, Km, and Cmin) for P as a function of the supply of Cu, Fe, Mn, and Zn, as well as the kinetic parameters of Cu, Fe, Mn, and Zn as a function of P supply in cotton (*Gossypium hirsutum* L.). The experiment was conducted in a greenhouse at the experimental unit of CENA, in Piracicaba, São Paulo, Brazil, using individual pots. Phosphorus concentration and accumulation were reduced only under Fe and Zn deficiency, with reductions of up to 60% in the shoots and 85% in the roots. Zn deficiency caused a drastic reduction in P uptake capacity, with Vmax decreasing from 590 to 50.85 µmol g^−1^ h^−1^ (approximately a 12-fold reduction), accompanied by an increase in Cmin (from 269 to 1508 µmol L^−1^). In terms of micronutrient kinetics, P omission reduced plant growth and affected only Fe and Zn uptake. For Fe, Km increased from 12.82 to 27.31 µmol L^−1^ and Cmin from 1.03 to 20.51 µmol L^−1^. For Zn, and Vmax decreased from 0.16 to 0.02 µmol g^−1^ h^−1^ (approximately 8-fold), while Cmin increased from 0.08 to 1.56 µmol L^−1^. These results demonstrate a strong interaction between P, Fe, and Zn, highlighting their regulatory roles in nutrient uptake and providing mechanistic insights into plant nutritional efficiency.

## 1. Introduction

Phosphorus (P) availability is a major constraint to crop productivity worldwide because of phosphorus’s low mobility in soil and strong sorption to mineral surfaces [1,2,3,4,5,6]. Thus, due to the low recovery of P by plants when supplied via phosphate fertilizers, high doses of P are applied annually to ensure optimal production. However, imbalances in nutrient supply may lead to deficiencies of other elements, particularly micronutrients such as zinc (Zn), especially in soils with inherently low Zn availability [7,8,9,10].

Nutrient imbalance can trigger physiological and biochemical responses that alter the uptake of other elements. For example, high levels of phosphorus (P) available to plants may lead to reduced zinc (Zn) uptake, an interaction widely discussed in the literature [2,11,12,13]. In this context, while deficiencies of micronutrients such as Zn may affect P acquisition, P deficiency has also been associated with changes in the uptake of several micronutrients. Although these responses are often discussed with a focus on the P–Zn interaction, the effects of individual nutrient limitations on the uptake of other nutrients have also been evaluated indirectly.

The mechanisms underlying these responses are not yet fully understood; however, nutrient availability to plants may involve changes in transporter activity, nutrient affinity, and internal distribution processes, which ultimately affect nutrient uptake efficiency [14,15,16,17]. Although the existence of such interactions is recognized, the literature lacks detailed descriptions of the kinetic parameters of P and micronutrient uptake as influenced by these interactions.

Previous studies have shown that P availability can influence the uptake of copper (Cu), Fe and manganese (Mn) and that micronutrient deficiencies may also modify P uptake dynamics [11,18,19,20,21,22,23]. In addition, it is important to elucidate these responses using an uptake kinetic approach. The correlation between P and Zn availability and the kinetic parameters of P and metal uptake contributes to a better understanding of the knowledge gap regarding the P–Zn interaction.

The kinetic parameters of nutrient uptake (Vmax, Km, and Cmin), as described by the Michaelis–Menten model, represent the following: Vmax corresponds to the product of the number of nutrient transporters per unit area of the plasma membrane and the rate at which these transporters operate within the membrane; Km represents the inverse of the transporter’s affinity for the nutrient and Cmin is the concentration at which the root is no longer able to absorb the nutrient [24].

According to the Michaelis–Menten model, a high Vmax and low Km and Cmin values characterize efficient nutrient uptake. However, when comparing different conditions, it is also useful to describe the uptake efficiency (α), which is calculated from the ratio between Vmax and Km [25]. In this way, the effect of the P–Zn interaction on these indices describes how this interaction interferes with the uptake dynamics of these elements at the molecular level, specifically the active transport of P and Zn in roots [15,16,26]. Thus, it is hypothesized that P availability influences the uptake kinetics of metals (Cu, Fe, Mn and Zn) through the induction of a high-affinity transporter system, and the opposite may also be true. Therefore, using the uptake kinetic technique, the objective of the present study was to compare the kinetic parameters of Cu, Fe, Mn and Zn uptake (Vmax, Km and Cmin) as a function of P availability, as well as the kinetic parameters of P uptake as a function of Zn availability.

Considering the existence of controversial and fragmented information regarding how nutrient availability affects the uptake of other elements, it is hypothesized that P availability may alter the uptake kinetics of cationic micronutrients such as Cu, Fe, Mn, and especially Zn and/or vice versa. Thus, this study aimed to evaluate, through uptake kinetic parameters, the dynamics of P absorption as a function of the supply of cationic metals, as well as the kinetic parameters of metal uptake as a function of P supply, in cotton.

## 2. Results

### 2.1. Kinetic Parameters in P Uptake

**Figure 1:**
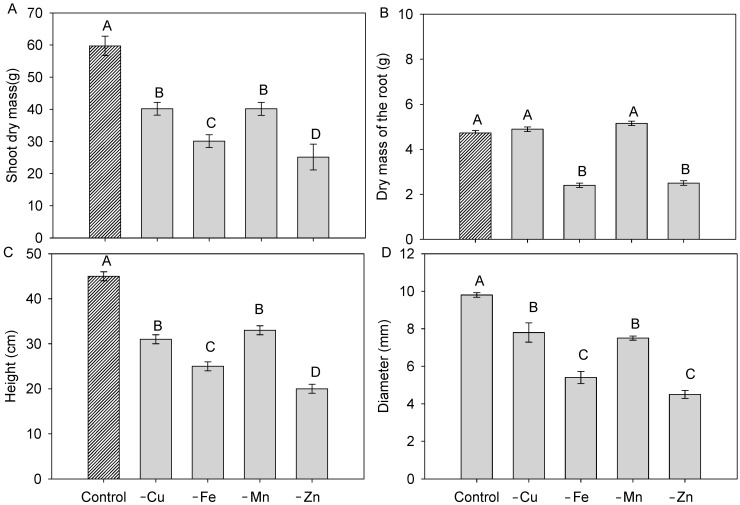
Shoot dry mass (**A**), root dry mass (**B**), height (**C**), and diameter (**D**) of cotton plants grown in complete nutrient solution and with total omission of copper (Cu), iron (Fe), manganese (Mn), and zinc (Zn) during the full flowering stage. The bar with a different texture refers to the control treatment. Bars represent mean ± standard error of the mean (SEM). Means followed by the same letter do not differ according to Tukey’s test (*p* ≤ 0.05).

The individual omissions of Cu, Fe, Mn, and Zn in the nutrient solution generally reduced shoot dry mass production by up to 58% (Zn), compared with plants grown in complete nutrient solution (Figure 1A). For root dry mass, only the omission of Fe and Zn resulted in reduced root system growth. Plants grown with total omission of Fe and Zn showed a reduction in root dry mass of approximately 60% compared with plants grown in complete nutrient solution (Figure 1B). In contrast, plant height and stem diameter followed a general pattern similar to shoot biomass, showing consistent decreases under all micronutrient omissions, although with varying magnitudes—being more pronounced under Fe and Zn omission (Figure 1C,D).

**Figure 2:**
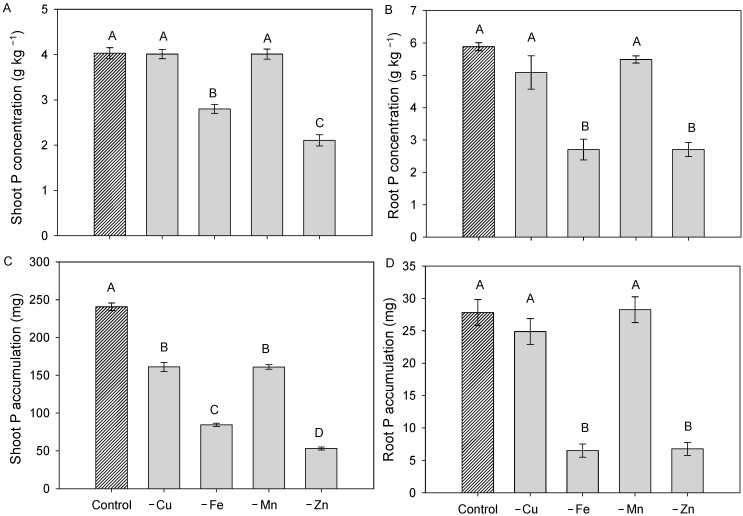
Shoot (**A**) and root P concentration (**B**) and Shoot (**C**) and Root P accumulation (**D**) in the dry weight of cotton plants grown in complete nutrient solution and with total omission of copper (Cu), iron (Fe), manganese (Mn), and zinc (Zn) during the full flowering stage. The bar with a different texture refers to the control treatment. Bars represent mean ± standard error of the mean (SEM). Means followed by the same letter do not differ according to Tukey’s test (*p* ≤ 0.05).

Phosphorus concentration in both shoots and roots was reduced under Fe and Zn omission, with decreases of approximately 40% and 60%, respectively, compared with plants grown in complete nutrient solution (Figure 2A). Quite the reverse, the omission of Cu and Mn did not markedly affect P concentration. Overall, these results indicate that Fe and Zn play a more prominent role in regulating root development and P nutritional status in cotton.

Regarding P concentration in the roots, Fe- and Zn-deficient plants showed a reduction of approximately 50% compared to plants grown in a complete nutrient solution (Figure 2B). Shoot P accumulation followed a pattern similar to that observed for shoot dry mass. There were reductions in all micronutrient omissions, particularly in Fe deficiency, which showed the greatest decrease (approximately 60%), while the other elements showed more moderate reductions, compared to plants grown in complete nutrient solution (Figure 2C).

For root P accumulation, only plants grown in solution with the individual omission of Fe and Zn showed lower P accumulation in the roots. Plants grown with total omission of Fe and Zn showed a reduction in root P accumulation of approximately 85% compared to plants grown in a complete nutrient solution (Figure 2D).

**Figure 3:**
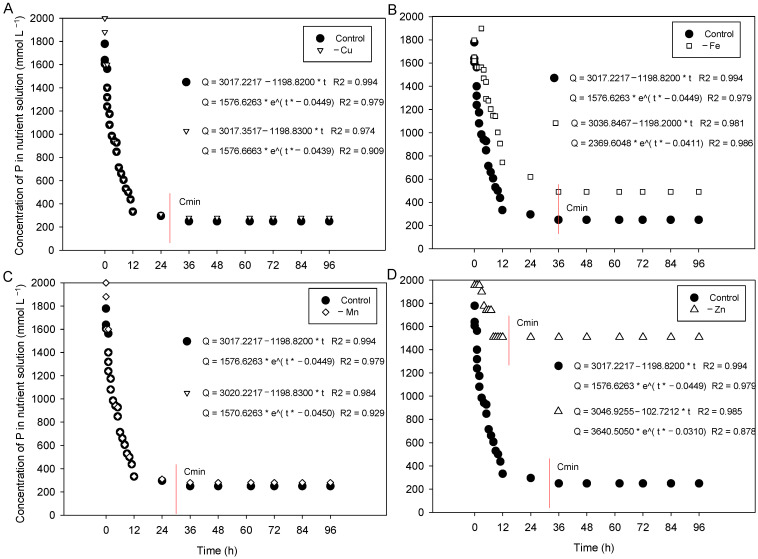
Phosphorus depletion curves as a function of the omission of Cu (**A**), Fe (**B**), Mn (**C**) and Zn (**D**) or presence (Control) of cations in plants grown in nutrient solutions with P over time (h).

The P depletion curve in the nutrient solution showed an exponential fit regardless of the concentration of Cu, Fe, Mn, or Zn (Figure 3). At all concentrations of Cu, Fe, Mn, and Zn, the Cmin of P in the solution was reached after approximately 24 h of exposure of the cotton plants to the treatments. However, the total omission of Cu or Mn in the nutrient solution did not affect P uptake by cotton plants, as the graphical model of the two curves (complete vs. metal omission) was very similar. On the other hand, plants deficient in Fe and Zn showed reduced P uptake capacity.

The kinetic parameters (Vmax, Km, Cmin, and α) were significantly affected by the availability of Fe and Zn in the nutrient solution (Table 1). Plants grown with omission of Zn showed a Vmax approximately 12 times lower than that of plants grown in a complete nutrient solution. In addition, Zn-deficient plants showed reduced Km, increased Cmin, and reduced uptake efficiency (α) compared to plants grown in a complete nutrient solution. Another limiting nutrient for P uptake was Fe. The omission of Fe also reduced Km and increased Cmin in cotton plants. These results indicate a lower capacity of Fe- and Zn-deficient plants to absorb P, although through different mechanisms.

### 2.2. Kinetic Parameters in Metal Uptake

**Figure 4:**
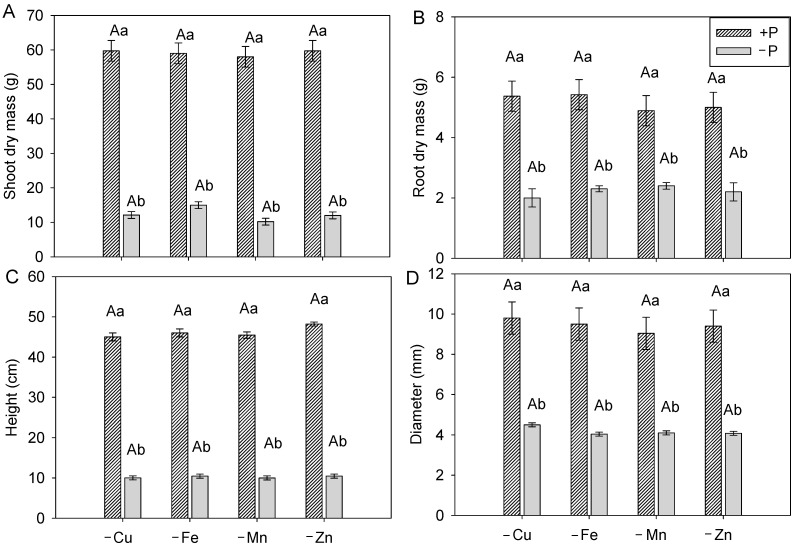
Shoot dry mass (**A**), root dry mass (**B**), plant height (**C**), and stem diameter (**D**) of cotton plants grown in complete nutrient solution (+P) and with total omission of phosphorus (−P) during the full flowering stage. Lowercase letters compare micronutrients within each P condition (+P or −P), while uppercase letters compare P supply within each micronutrient. Bars represent mean ± standard error of the mean (SEM). Means followed by the same letter do not differ according to Tukey’s test (*p* ≤ 0.05).

The omission of P in the nutrient solution reduced shoot and root dry mass production, as well as plant height and stem diameter, compared with plants grown in complete nutrient solution (Figure 4).

**Figure 5:**
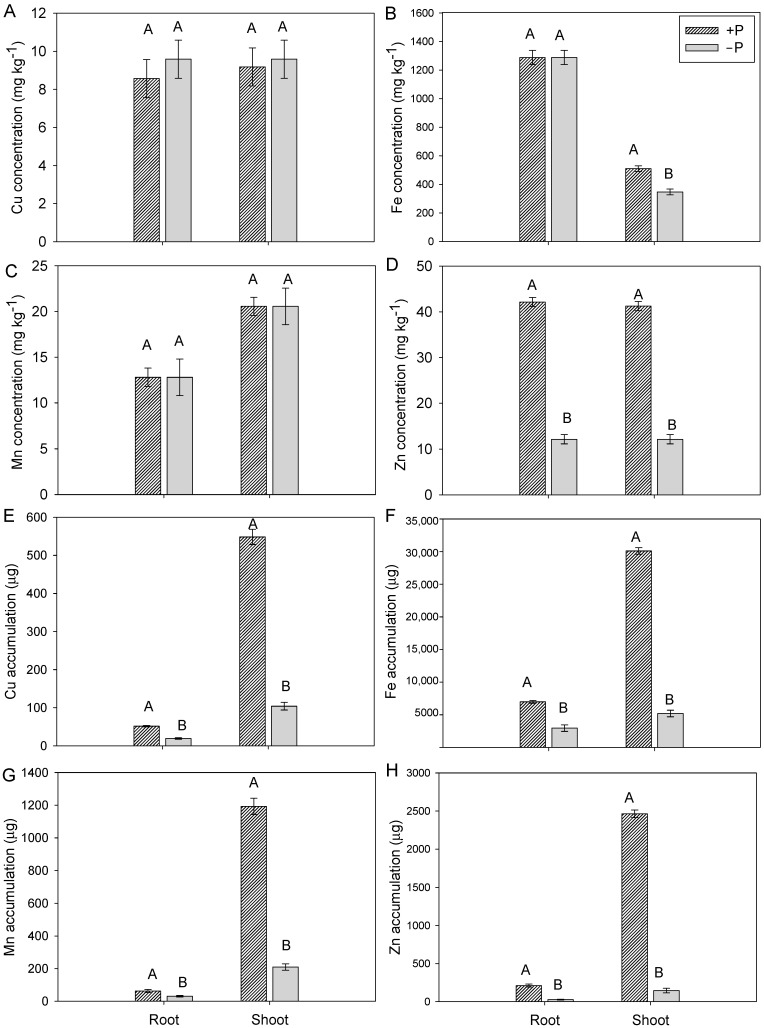
Concentration of copper (Cu) (**A**), iron (Fe) (**B**), manganese (Mn) (**C**), and zinc (Zn) (**D**); and accumulation of Cu (**E**), Fe (**F**), Mn (**G**), and Zn (**H**) in the dry weight of the shoot and root of cotton plants grown in complete nutrient solution (+P) and under total omission of phosphorus (−P) during the full flowering phase. Bars represent mean ± standard error of the mean (SEM). Means followed by the same letter do not differ according to Tukey’s test (*p* ≤ 0.05).

Thus, P omission in the nutrient solution induced reduced growth of cotton plants, regardless of the metal omitted for the kinetic assay. Plants grown with adequate P supply (+P) showed no differences in growth, and the same pattern was observed among plants grown without P (−P). The concentrations of Cu, Fe, and Mn in the roots of cotton plants were not influenced by P supply in the nutrient solution (Figure 5A–C).

In the shoot, P supply also did not influence Cu and Mn concentrations, similar to what was observed in the roots. On the other hand, plants grown with P omission showed reduced Fe concentration in the shoot (Figure 5C). The concentrations of Zn were reduced by the total omission of P in the growth solution, both in roots and shoots (Figure 5D).

Regarding the accumulation of these metals in cotton plants, the omission of P in the nutrient solution reduced the accumulation of Cu, Fe, Mn, and Zn in both shoot and root tissues (Figure 5E–H), similarly to what was observed for shoot and root dry mass.

**Figure 6:**
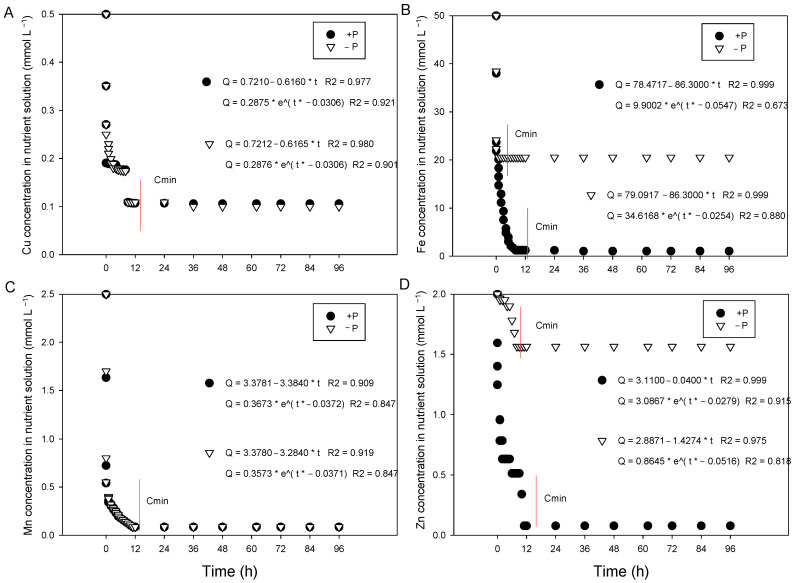
Depletion curves of Cu (**A**), Fe (**B**), Mn (**C**) and Zn (**D**) as a function of phosphorus omission (−P) or presence (+P) in plants grown in nutrient solution over time (h).

The depletion curves of Cu, Fe, Mn, and Zn in the nutrient solution showed an exponential fit, regardless of the P concentration in the nutrient solution (Figure 6). In all depletion curves, Cmin in the solution was reached after approximately 12 h of exposure of cotton plants to the treatments. However, the total omission of P in the growth solution affected only the uptake of Fe and Zn by cotton plants.

The omission of P in the growth solution did not influence the kinetic parameters of Cu and Mn uptake in cotton (Table 2). However, the effect of P on the kinetic parameters of Fe and Zn uptake was observed. P-deficient plants showed increased Cmin, as well as reduced Km and uptake efficiency (α), compared with plants grown in a complete nutrient solution. When analyzing the kinetic parameters of Zn uptake, it was observed that P deficiency also induced an increase in Cmin and a reduction in Km and uptake efficiency (α), compared to plants grown in complete nutrient solution. In addition, P deficiency also led to a reduced Zn Vmax in the nutrient solution.

## 3. Discussion

The results demonstrate that the interaction between phosphorus and micronutrients is not uniform, revealing a selective effect of Fe and Zn on P uptake kinetics. While the omission of Mn and Cu caused only limited or inconsistent changes in kinetic parameters, Fe and especially Zn deficiency led to pronounced reductions in Vmax, indicating a decrease in the maximum uptake capacity of the root system. These findings suggest that P acquisition efficiency is more strongly associated with the availability of specific micronutrients rather than with a generalized micronutrient effect. The consistent response observed for Fe and Zn reinforces the interpretation that these elements play a more relevant physiological role in modulating nutrient uptake efficiency, possibly due to their involvement in membrane transport activity, root metabolic processes, and maintenance of functional uptake systems. Thus, the kinetic approach adopted in this study contributes to a more integrated understanding of nutrient interactions by demonstrating that the regulation of P uptake efficiency depends on specific micronutrient conditions rather than on a broad micronutrient limitation effect.

In the P uptake kinetic study, the results of Cu, Fe, Mn, and Zn concentrations showed that plants grown with individual omission of each metal were deficient in the omitted element, since the concentration of these nutrients plant organs was below the adequate level [27]. Thus, the study compared plants deficient in Cu, Fe, Mn, or Zn with healthy plants. The individual omissions reduced the growth of cotton plants grown under adequate P concentration. This result was expected since all these micronutrients are highly correlated with cotton growth rate [22]. Most of the Cu in leaf cells is associated with plastocyanin, which participates in electron flow in the light phase of photosynthesis, linking the two photosystems. Cu also participates in the dark phase of photosynthesis by activating ribulose bisphosphate carboxylase, responsible for the incorporation of CO_2_ into organic compounds. Mn, in turn, is the ion responsible for water photolysis during the photochemical phase of photosynthesis [28].

The differential responses observed in Vmax and Km provide important biological insights into how micronutrient availability influences phosphorus uptake in cotton. Changes in Vmax are generally associated with variations in the maximum uptake capacity of the root system, reflecting alterations in transporter density or activity at the plasma membrane. In contrast, variations in Km are related to the affinity of transport systems for the nutrient, indicating modifications in the efficiency with which roots perform P uptake under low external concentrations. In the present study, the pronounced reduction in Vmax under Zn and Fe deficiency suggests a limitation in the capacity of cotton roots to perform P uptake, possibly due to metabolic constraints affecting membrane transport activity or root physiological performance. Meanwhile, changes observed in Km indicate that micronutrient supply may also influence the efficiency of P uptake under limiting conditions, which is particularly relevant in agricultural systems where P availability is naturally low. These results indicate that Zn and Fe availability affects not only the amount of P taken up but also the efficiency of the uptake process, reinforcing the physiological importance of balanced micronutrient supply for optimizing P use efficiency in cotton.

Fe is closely associated with dry mass production because plants deficient in Fe show low respiratory activity due to impairment in electron transport during the terminal oxidations occurring in mitochondria. In addition, Fe participates in several enzymes involved in nitrogen and sulfur metabolism, such as nitrate reductase, nitrite reductase, sulfite reductase, and nitrogenase [28,29]. Zn, on the other hand, is required at the active site of carbonic anhydrase, an enzyme responsible for the fixation of atmospheric CO_2_. Carbonic anhydrase located in the cytoplasm and chloroplast may facilitate carbon transfer for fixation and carbohydrate synthesis [28].

Only plants deficient in Fe and Zn showed reduced P concentration in the shoot and root, even though P was present at adequate concentrations in the nutrient solution (2 mmol L^−1^). However, when P accumulation was evaluated, all omissions reduced P accumulation in cotton plants. This result suggests that the lower dry mass production observed in plants grown with metal omission [30,31,32]. Thus, all omissions of Cu, Fe, Mn, and Zn caused a reduction in P accumulation, but only Fe and Zn omissions altered the P uptake capacity of cotton plants. These finding highlights that, beyond general nutritional effects, Fe and Zn specifically regulate P uptake processes, which is further supported by the kinetic approach adopted in this study. The results of the uptake kinetic study confirm this hypothesis. However, it is noteworthy that Fe deficiency modifies P uptake differently from Zn deficiency.

Fe-deficient plants showed a affected the affinity component Km, implying a reduction in the overall capacity for P uptake (higher Cmin). The reduction in Km implies lower activity of high-affinity transporters (HATs—High Affinity Transport System) [25], which is reflected in a lower capacity for nutrient uptake over time. However, Vmax was not influenced by Fe deficiency. Thus, it is believed that Fe deficiency reduces the expression of HATs but does not affect Vmax. Even so, the reduction in Km alone is sufficient to reduce the P uptake capacity. Although P–Fe interactions have been reported in several agricultural crops [11,13,15,19,23], these studies have primarily described physiological or antagonistic effects, with limited insight into the underlying uptake mechanisms. In this context, the present study advances current knowledge by demonstrating, through kinetic parameters, that Fe availability specifically modulates P uptake by affecting transporter affinity rather than transport capacity.

Fe is an essential micronutrient for plant cell function, acting as a cofactor in metabolic processes, particularly during photosynthesis; therefore, it is not surprising that P or Fe deficiency inhibits plant growth. Many studies have analyzed the effects of P and/or Fe deficiency in rice at the molecular level. For example, a global gene expression analysis using microarray experiments was performed to investigate transcriptome changes in rice in response to Fe or P alterations and to explore the interactions between Fe and P signaling in rice [33]. The results of this study showed that most transcriptional changes observed under Fe deficiency were repressed when P was also absent (−Fe–Pi). At the phenotypic level, P and Fe were shown to interact antagonistically in modulating rice growth [34,35,36]. For example, rice growth is severely affected by Fe deficiency but can be partially reversed by simultaneous deficiencies of Fe and P. Beyond rice, an interaction between P and Fe signaling pathways has been observed in many plant species and has perhaps been best studied in the model plant *Arabidopsis thaliana* [37]. Thus, it is evident that further studies addressing the nutritional balance related to the complex P–Fe interaction over time are needed, using different tools and methodologies for plant nutritional diagnosis.

On the other hand, Zn deficiency modified all kinetic parameters of P uptake. Thus, Zn not only reduced the activity of high-affinity transporters (HATs—High Affinity Transport System), an important response under low P supply conditions, such as those imposed 24 h before the kinetic study, but also influenced the uptake capacity (Vmax) and P uptake efficiency (α). Unlike Fe, which primarily affected transporter affinity, Zn deficiency altered both affinity and maximum uptake capacity, indicating a broader regulatory role in P acquisition. Based on these results, it can be stated that Zn plays an important role in signaling P deficiency in cotton, since all kinetic parameters of P uptake were negatively affected by Zn deficiency.

The P–Zn interaction in cotton plants has already been reported by [4]. However, previous studies have mainly focused on changes in nutrient concentration and accumulation, without addressing the kinetic basis of this interaction. The results of P concentration and accumulation demonstrated the control of P uptake by Zn. Under low Zn supply, plants showed high P accumulation and high P concentration in the shoot. Under normal growth conditions, P uptake by plants is strictly regulated to maintain P concentration within physiological limits and maintain homeostasis [38,39,40]. However, this control is reduced under Zn deficiency, leading to P accumulation [41,42,43]. Zn plays a specific role in the signal transduction pathway responsible for regulating genes that encode P transporters [41,44]. Ref. [39] reported that Zn deficiency specifically signals an increase in the expression of high-affinity P transporters in the roots, regardless of P availability in the growth medium. In addition to increasing the expression of these transporters in the roots, Zn deficiency also increases the expression of P transporters responsible for loading P into the xylem, consequently contributing to P accumulation in the shoot [40]. Thus, cotton plants deficient in Zn did not efficiently regulate P uptake from the nutrient solution.

Adequate Zn supply in the nutrient solution reduced the concentration and accumulation of P in the shoot. This reduction in P uptake is associated with increased Zn accumulation in the roots, which induces the plant to absorb less P [41,45,46,47]. However, this effect was observed at higher P doses, indicating the beneficial role of Zn in controlling P uptake under conditions of high P availability. The results of shoot dry mass confirm this hypothesis, as the highest dry mass accumulation was found in plants grown with the highest P and Zn doses.

The present study suggests that Fe and Zn increase P uptake, probably by allocating this nutrient to the cells where it is most needed. Importantly, by integrating nutrient omission with uptake kinetics, this study provides mechanistic evidence that the P–Fe and P–Zn interactions are mediated by changes in transporter behavior rather than solely by changes in nutrient availability or plant growth. Molecular mechanisms may stimulate the efficient use of these two nutrients through signaling and regulatory networks. For sustainable agriculture worldwide, it is necessary to encourage studies that improve the understanding of P–Zn and P–Fe signaling.

In the metal uptake kinetic study, the results of P concentration showed that plants grown with total omission of P in the nutrient solution were P-deficient, since the concentration of this nutrient in plant organs was below the adequate level [27]. Unlike what was observed in the P kinetic study, where Zn or Fe deficiency increased Km, in the Fe and Zn kinetic study, P deficiency increased Km. Thus, P deficiency increased the activity of high-affinity transporters (HATs) under deficiency conditions. These contrasting responses reinforce the bidirectional nature of the interaction, highlighting that P not only is regulated by micronutrients but also actively modulates the uptake kinetics of Fe and Zn.

## 4. Materials and Methods

### 4.1. Experimental Design, Characterization, and Development

To achieve the objectives of this study, an experiment was conducted in a greenhouse at the Centro de Energia Nuclear na Agricultura of the Universidade de São Paulo, located in Piracicaba, São Paulo, Brazil, using cotton plants (*Gossypium hirsutum* L.) of the cultivar FMT 709.

During the experimental period, plants were maintained in a greenhouse under controlled environmental conditions. The average air temperature was maintained at 28 ± 3 °C during the day and 20 ± 2 °C at night, with relative humidity ranging from 60 to 75%. The photoperiod followed natural light conditions typical of the region (approximately 12–13 h of light), with average photosynthetically active radiation (PAR) at canopy level ranging from 600 to 900 µmol m^−2^ s^−1^ at midday.

The seeds were germinated in shallow trays containing vermiculite moistened with a calcium sulfate solution (CaSO_4_, 10^−4^ mol L^−1^). When the seedlings reached approximately 5 cm in height, they were transferred to plastic trays with a capacity of 40 L containing nutrient solution [48], complete and diluted to one-fifth of the usual concentration and referred to as the “adaptation solution” in order to intensify nutritional deficiencies while accounting for the contribution of seed reserves. After one week in this adaptation solution, the plants were transferred to plastic pots with a capacity of 3.5 L containing 3.0 L of nutrient solution, thus initiating the treatments.

For the P uptake kinetic study, the plants were grown in complete nutrient solution (control) and in a nutrient solution with total omission of Cu, Fe, Mn, and Zn in order to evaluate the effect of the omission of these cationic micronutrients on the dynamics of P uptake by cotton. For the uptake kinetic study of Cu, Fe, Mn, and Zn, the plants were grown in complete nutrient solution (+P) and in nutrient solution with total omission of P (−P). Thus, a completely randomized design with four replications was used for each kinetic experiment (P kinetics and metal kinetics).

All chemical reagents used in the preparation of the nutrient solutions were of analytical grade. Macronutrients were supplied using the following salts: calcium nitrate tetrahydrate (Ca(NO_3_)_2_·4H_2_O), potassium nitrate (KNO_3_), ammonium dihydrogen phosphate (NH_4_H_2_PO_4_), magnesium sulfate heptahydrate (MgSO_4_·7H_2_O), and potassium chloride (KCl). Micronutrients were supplied as iron EDTA (Fe-EDTA), boric acid (H_3_BO_3_), manganese chloride tetrahydrate (MnCl_2_·4H_2_O), zinc sulfate heptahydrate (ZnSO_4_·7H_2_O), and copper sulfate pentahydrate (CuSO_4_·5H_2_O). The complete nutrient solution had the following composition: 12.0 mmol L^−1^ N–NO_3_^−^; 4 mmol L^−1^ N–NH_4_^+^; 2.0 mmol L^−1^ P; 6.0 mmol L^−1^ K; 4.0 mmol L^−1^ Ca; 2.0 mmol L^−1^ Mg; 2.0 mmol L^−1^ S; 50.0 µmol L^−1^ Cl; 50 µmol L^−1^ Fe; 25.0 µmol L^−1^ B; 2.5 µmol L^−1^ Mn; 2.0 µmol L^−1^ Zn; and 0.5 µmol L^−1^ Cu. In the treatment solutions with omission of P, B, Cu, Mn, and Zn, the concentrations were identical to those of the complete solution except for the nutrient under study.

The pH of the nutrient solution was monitored daily using a digital pH meter and maintained at 5.5 ± 0.2 throughout the experimental period by adjustment with diluted HCl or NaOH solutions when necessary. Maintaining a stable pH was essential to ensure adequate nutrient availability and to avoid changes in the chemical speciation of micronutrients, particularly cationic metals such as Fe, Zn, Mn, and Cu, whose solubility and uptake are strongly influenced by pH variations.

The nutrient solution was renewed every 14 days to prevent nutrient imbalance and excessive pH drift resulting from plant uptake processes. The use of controlled pH conditions ensured the stability of the ionic environment of the solution and allowed for the reliable determination of nutrient uptake kinetic parameters. The pH range adopted is widely considered adequate for hydroponic cultivation of cotton and for maintaining the availability of phosphorus and micronutrients in solution.

The plants were fixed at the collar region with plastic foam and maintained under constant aeration using an air pump system to ensure adequate oxygen supply to the nutrient solution. During the experimental period, the nutrient solutions were renewed approximately every 14 days, and the volume in the pots was replenished daily with deionized water when necessary.

### 4.2. Uptake Kinetics

When the plants reached the stage of first floral bud emission (70 days after emergence—DAE), the determination of uptake parameters was carried out based on the uptake of P or metals (Cu, Fe, Mn, and Zn) from the depletion solution as a function of time, as described by [49]. Only the nutrient under study was omitted from the depletion solution for 24 h while maintaining the treatments. Thus, for the micronutrient uptake kinetic study, either Cu, Fe, Mn, or Zn was individually omitted from the solution of plants grown in nutrient solution with P (+P) and without P (−P). For the P uptake kinetic study, the phosphate supply was omitted (−P). After 24 h in the depletion solution, the plants were transferred to a nutrient solution containing the same concentration of P, Cu, Fe, Mn and Zn as the previous solution. The solution was constantly aerated using an air compressor and the evapotranspired volume was replenished by adding deionized water. After 30 min, sufficient time for the plants to reach a steady state [50], solution sampling began. Samples of 10 mL were collected every 15 min during the first two hours; every 30 min during the following four hours; every 60 min during the subsequent six hours; and finally, every 12 h until the concentrations of P and micronutrients became constant, totaling 96 h of sampling. The volume in the pots was kept constant by replenishing the equivalent sampled volume with deionized water. The determination of P, Cu, Fe, Mn, and Zn in the solution samples was performed according to [17]. After the depletion experiment, the plant roots were collected and dried in a forced-air oven (±65 °C), followed by the determination of root dry mass.

The concentration data from the depletion solution at each sampling time, as well as the data related to the solution volume in the pots, total sampling time, sample volume, and root dry mass, were used to calculate the kinetic parameters Km and Vmax for each replication using the software CinéticaWin version 1.2 (Brazil) [51]. The Cmin was estimated as the average concentration of P and micronutrients in the solution from the point at which their concentrations tended to remain constant, and α (uptake efficiency) was calculated as the ratio between Vmax and Km.

### 4.3. Dry Matter Production, Plant Height, Stem Diameter, and Mineral Analysis of Shoots and Roots

At the end of the experiment, plants were harvested and separated into shoots and roots. The roots were carefully removed from the pots and rinsed with deionized water to remove residual nutrient solution adhering to the root surface. When necessary, a rapid desorption procedure was performed to minimize superficial contamination by ions adsorbed to the root apoplast before drying. The plant material was then identified, placed in paper bags, and dried in a forced-air oven at 65 °C for 72 h until constant weight.

After drying, shoot and root samples were weighed separately and ground in a Wiley-type mill equipped with a 1 mm mesh sieve. For mineral determination, the ground plant material was subjected to nitric-perchloric digestion according to the methodology described by Malavolta et al. [17]. Phosphorus concentration was determined by colorimetry, whereas Cu, Fe, Mn, and Zn concentrations were quantified by atomic absorption spectrophotometry. The results were expressed on a dry matter basis for each plant organ (shoots and roots). Nutrient accumulation was calculated by multiplying nutrient concentration by the corresponding dry mass of each organ [4].

### 4.4. Statistical Analysis

The results were subjected to statistical analysis using SAS statistical software [52] at a significance level of 5%. Data were tested for normality and homogeneity of variance prior to analysis. The results were subjected to analysis of variance (ANOVA). When significant effects were observed, means were compared using Tukey’s test at *p* ≤ 0.05. Data are presented as mean ± standard error of the mean (SEM). Kinetic parameters (Vmax, Km, Cmin, and α) were estimated for each replicate using CinéticaWin software version 1.2 (Brazil), and the obtained values were subjected to ANOVA followed by Tukey’s test (*p* ≤ 0.05).

## 5. Conclusions

Phosphorus uptake in cotton was influenced by the omission of Fe and Zn in the nutrient solution, with Zn deficiency reducing both the P uptake rate and the capacity for expression of high-affinity transporters. In the study, P omission affected only Fe and Zn uptake. These findings suggest that P, Fe, and Zn interactions affect the physiological mechanisms associated with nutrient uptake.

The combination of transcriptomics and metabolomics may be an effective tool for clarifying the cross-talk between P, Fe, and Zn metabolic pathways and for identifying metabolites that may play negative and/or positive regulatory roles in the co-regulation of plant nutrition. Developing a comprehensive understanding of the P × Fe × Zn signaling interaction will be of great scientific interest and crucial for sustainable agriculture worldwide.

## Figures and Tables

**Table 1:** Kinetic parameters Vmax (maximum uptake rate), Km, Cmin, and α for P uptake in plants deficient in Cu, Fe, Mn, and Zn.

Treatment	Vmax	Km	Cmim	α
	µmol/g·h	µmol/L	µmol	
Control	590.01 ± 13.22 a	1226.66 ± 15.17 a	269.38 ± 14.13 c	0.600 ± 11.03 a
Omission of Cu	593.17 ± 11.31 a	1120.08 ± 22.13 a	259.23 ± 12.12 c	0.527 ± 12.11 a
Omission of Fe	580.02 ± 48.22 a	779.77 ± 48.22 c	490.25 ± 13.13 b	0.708 ± 14.10 a
Omission of Mn	595.11 ± 10.01 a	1125.80 ± 20.13 a	270.28 ± 30.13 c	0.591 ± 12.13 a
Omission of Zn	50.85 ± 12.03 b	928.26 ± 10.15 b	1508.28 ± 30.14 a	0.055 ± 13.11 b

Data are presented as mean ± standard error of the mean (SEM). Different letters indicate significant differences according to Tukey’s test (*p* ≤ 0.05).

**Table 2:** Kinetic parameters Vmax (maximum uptake rate), Km, Cmin, and α for P uptake in Cu-deficient plants.

Treatment	Vmax	Km	Cmim	α
	µmol/g·h	µmol/L	µmol	
	Cu kinetics			
Control	0.0048 ± 0.00001 a	0.09 ± 0.001 a	0.11 ± 0.01 a	0.053 ± 0.002a
Omission of P	0.0048 ± 0.00001 a	0.09 ± 0.002 a	0.10 ± 0.01 a	0.050 ± 0.001 a
	Fe kinetics			
Control	42.72 ± 0.01 a	12.82 ± 0.02 b	1.03 ± 0.03 b	3.33 ± 0.02 a
Omission of P	42.72 ± 0.02 a	27.31 ± 0.02 a	20.51 ± 0.02 a	1.56 ± 0.04 b
	Mn kinetics			
Control	1.63 ± 0.03 a	12.89 ± 0.4 a	0.66 ± 0.01 a	0.127 ± 0.02 a
Omission of P	1.64 ± 0.01 a	12.80 ± 0.04 a	0.66 ± 0.01 a	0.126 ± 0.01 a
	Zn kinetics			
Control	0.16 ± 0.02 a	0.32 ± 0.01b	0.08 ± 0.02 b	0.10 ± 0.02 a
Omission of P	0.02 ± 0.01 b	0.87 ± 0.01 a	1.56 ± 0.03 a	0.02 ± 0.01 b

Data are presented as mean ± standard error of the mean (SEM). Different letters indicate significant differences according to Tukey’s test (*p* ≤ 0.05).

## Data Availability

The data supporting the conclusions of this article will be made available by the authors on request.
